# Splenic abscess complicated by pleural empyema: A rare case report from rural Nepal

**DOI:** 10.1016/j.ijscr.2020.09.145

**Published:** 2020-09-24

**Authors:** Suman Baral, Raj Kumar Chhetri, Milan Gyawali, Neeraj Thapa

**Affiliations:** Department of Surgery, Lumbini Medical College and Teaching Hospital Ltd, Tansen-7, Pravas, Palpa, Nepal

**Keywords:** Empyema thoracis, Splenic abscess, *Streptococcus pyogenes*

## Abstract

**Introduction:**

Splenic abscess generally occurs through hematogenous spread and typically follows endocarditis or seeding from contiguous sites of infection. This can be complicated by empyema thoracis. We present a rarer case of chronic alcoholic with splenic abscess along with empyema thoracis.

**Presentation of a case:**

A 39-year old alcoholic male presented with history of pain at left hypochondrium and difficulty breathing for 7 days. Abdominal examination revealed tenderness at left hypochondrium along with enlarged spleen and liver associated with decreased air entry of left chest. Chest X-ray showed complete white out lung field on left side. Contrast enhanced tomography abdomen and pelvis revealed splenic abscess involving lower pole of spleen along with peri splenic extension. Tube thoracostomy drainage on left chest was done followed by ultrasonography guided repeated aspiration of splenic entity. Pus culture sensitivity showed growth of *Streptococcus pyogenes* while splenic aspirate remained sterile. Patient got discharged on 8th day of admission with full recovery.

**Discussion:**

Splenic abscess is the rare entity which is commonly seen in immunocompromised individuals that might get complicated as empyema thoracis and management includes broad spectrum antibiotics along with tube thoracostomy and percutaneous drainage of splenic abscess if possible, in view of spleen being salvageable. Pleural collection revealed growth of *Streptococcus pyogenes* in our case which itself is the rare finding.

**Conclusion:**

Splenic abscess can be managed with percutaneous aspiration/drainage if lesion is unilocular in the view of salvaging spleen. Complicated empyema can be managed with tube thoracostomy along with broad spectrum antibiotics.

## Highlights

• Splenic abscess is one of the rarer findings which is commonly seen in immunocompromised individuals.
• Splenic abscess may present with features of pleural effusion along with empyema if gets infected.
• Pleural empyema needs tube drainage.
• Splenic entity if unilocular, can be managed with broad spectrum antibiotics along with percutaneous aspiration or drainage.
• Open drainage or splenectomy is mandatory in case percutaneous techniques fail.

## 1 Introduction

Splenic abscess is an uncommon entity encountered in clinical practice with overall incidence around 0.05–0.7% in autopsy studies [1]. It generally occurs through hematogenous spread and typically follows endocarditis or seeding from contiguous sites of infection [2]. Other risk factors include diabetes mellitus, congenital or acquired immunodeficiency, use of immunosuppressive drugs, intravenous drug abuse and hemoglobinopathies [3]. Owing to wide spread use of diagnostic modalities like USG and CECT, this uncommon disease is being diagnosed and managed these days [4]. Treatment includes medical management with antibiotics along with percutaneous aspiration. Splenectomy is warranted if the conservative approach fails [5,6]. We aim to articulate a male alcoholic patient who presented with pain left upper abdomen along with pleural empyema who was managed with tube thoracostomy and percutaneous drainage of the splenic entity. This work has been reported in accordance to the Surgical Case Report (SCARE) guidelines [7].

## 2 Presentation of a case

**Fig. 1:**
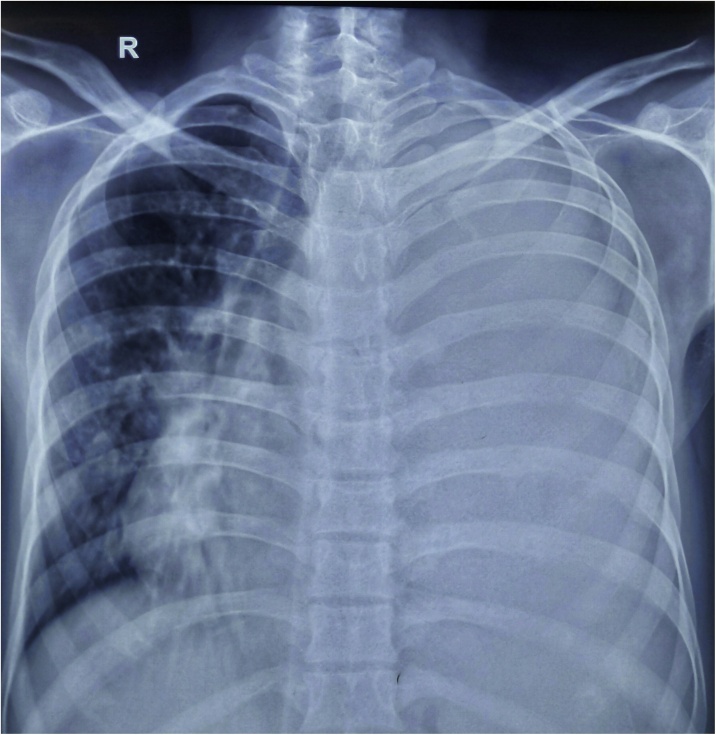
Showing the complete white out lung field on left chest with tracheal shift to right side suggestive of pleural effusion.

**Fig. 2:**
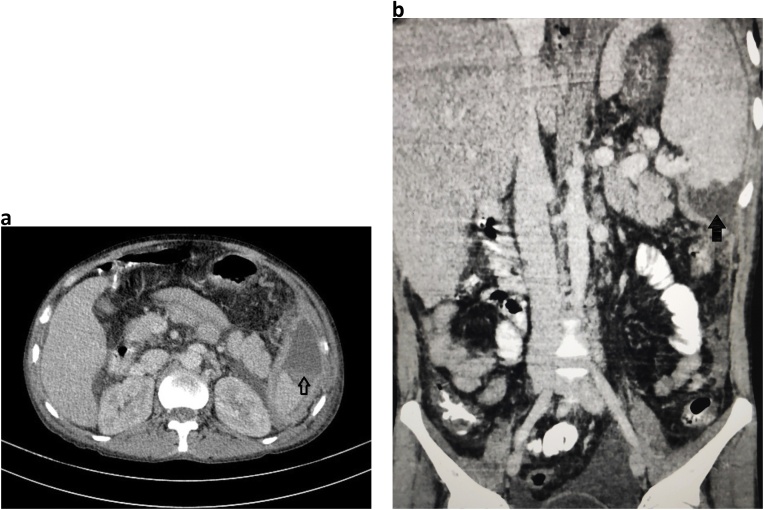
a: Axial section with arrow head showing splenic abscess involving lower pole of the spleen. b: Coronal section with blunt arrowhead reveals splenic abscess with peri splenic extension to the upper pole.

**Fig. 3:**
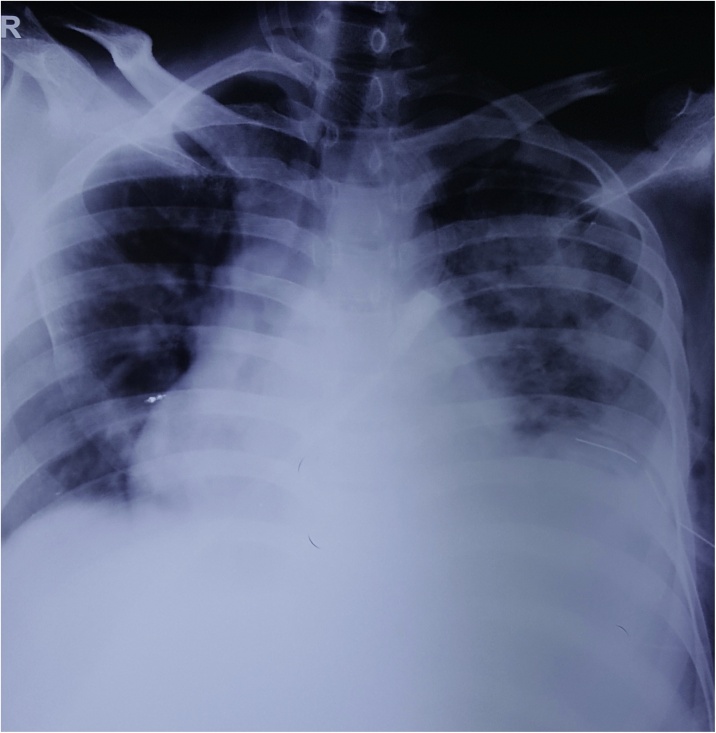
Shows the resolution of the white out lung field on left chest with tube in situ.

A 39-year-old male, chronic alcoholic presented to surgical outpatient department (OPD) with the chief complaints of pain in the left upper abdomen along with difficulty breathing for seven days. Pain was insidious in onset, dull aching type with no any relieving factors. Breathing difficulty was associated with productive cough, whitish in color, non-foul smelling and not mixed with blood. Cough was followed by lower chest pain in the left side. Also, there was a history of loose motion for four days, 3–4 times/day not mixed with blood or mucus. There was no history of fever, burning micturition, headache, dizziness, orthopnea or paroxysmal nocturnal dyspnea (PND). On examination, the patient looked ill. Icterus was present. Vitals were within normal limit except for respiratory rate (RR) which was 24 breaths/min. Spo2 was 92% in room air. Chest examination showed decreased air entry on left lung field with dullness appreciated while on percussion. Abdominal examination revealed tenderness on left hypochondriac region with splenomegaly around 3 cm below left subcoastal area along with enlarged liver around 7 cm below right subcoastal margin. Laboratory parameters showed hemoglobin (Hb) 10 gm/dl, Erythrocyte Sedimentation Rate (ESR)- 25 mm/1 st hour, Total Leucocyte Count (TLC)- 35,800/mm^3^ with neutrophilia (88%), prothrombin time (PT): 30 s with International Normalized Ratio (INR)- 2.07. Renal Function Test (RFT) was normal. HIV, HBSAG, HCV serology were negative. Stool examination along with WIDAL test was normal. Liver Function Test (LFT) showed total bilirubin 3.4 mg/dl, direct bilirubin 2.1 mg/dl, Alanine Amino Transferase (ALT) -15 U/L, Aspartate Amino Transferase (AST)- 40 U/L and Alkaline Phosphatase (ALP)- 216 U/L. Ultrasonography abdomen and pelvis showed enlarged liver and spleen with echogenic fluid noted in peri splenic region with no vascularity and low-level echoes floating within it. Free fluid was noted in the peritoneal cavity along with echogenic free fluid in left pleural space. Chest X ray showed complete white out lung field on left side (Fig. 1). Chest tube drain was inserted on the left chest, which drained around 1500 ml of pus. Contrast enhanced computed tomography (CECT) abdomen showed splenomegaly (14 cm) with non-enhancing hypodense collection of mean HU 14 measuring 6.3 × 3.3 × 5 cm (approx. 54 ml) in the inferior pole of the spleen with peri splenic extension along with irregularity at lower pole suggesting parenchymal origin of the abscess. Liver was enlarged (19 cm), portal vein diameter of 16 mm with left sided pleural effusion with collapse of posterior basal segment of left lung along with mild right sided pleural effusion and minimal pericardial effusion (Fig. 2a and b). Pleural aspirate analysis showed total cell count of 120,000/mm^3^ including leucocytes of 1,10,000/mm^3^ and red blood cells (RBC) 10,000/mm^3^ with polymorphs 85%, lymphocytes 15%, sugar 20 mg/dl, protein 3.2 gm/dl, albumin 2.3 gm/dl and fluid adenosine deaminase (ADA) of 90. Pleural fluid gram stain revealed few gram-positive cocci in pairs and chains whilst culture analysis showed *Streptococcus pyogenes* sensitive to ampicillin, azithromycin, cefotaxime and ceftriaxone. Tuberculosis workout including Mantoux, sputum acid fast bacilli (AFB) and gene expert from pleural fluid was negative. USG guided splenic abscess was aspirated, that revealed around 30 ml of pus initially followed by second aspiration of 10 ml the next day. Splenic abscess culture was sterile. The patient was managed with Ceftriaxone/Sulbactam along with Vancomycin and azithromycin according to culture sensitivity reports. Chest X ray gradually improved along with the condition of the patient (Fig. 3). Chest tube removal was done on 5th day of insertion. The patient got discharged on 8th day of admission.

## 3 Discussion

Splenic abscess which is uncommonly encountered during clinical practice has incidence of 0.05–0.7% in autopsy studies [1]. With the male preponderance, this clinical entity is most often seen in immunocompromised individuals [8]. During pre-antibiotics era, splenic abscess was encountered in patients with enteric fever however these days, the causes related are HIV/AIDS, urogenital infections, abdominal infections, bacterial endocarditis and respiratory illness like pneumonia [9]. Other causes include infections secondary to splenic infarction and traumatic injury to spleen [5]. A series from Italy which was reported on 16 patients, showed the 75% of their subjects to be males and 50% of the patients immunocompromised [4]. Immunodeficiency due to alcoholism, chronic liver disease and malignancies that have led to transplantation along with therapy with immunosuppressive have added the risk of development of splenic abscess [10]. Results from a review done on 287 patients showed immunocompromised status of patients in 33.5% cases which included nearly half with intravenous drug abuse along with HIV/AIDS [8].

The most common organisms involved in splenic abscess have been aerobic bacteria mostly *Streptococci* and *Escherichia coli,* however various literatures have suggested *Mycobacterium tuberculosis* and *Salmonella typhi* as the organisms responsible for abscess [8,11,12]. Fungal abscesses have been reported in immunocompromised individuals [13]. Chang et al. in their series of 67 cases in Taiwan over the period of 19 years found *Klebsiella pneumonia* as the most common cause of splenic abscess [5]. However, the splenic aspirate in our patient was sterile with no growth of organism.

The clinical symptoms and presentation can be variable depending upon the stage of presentation, location and size of the lesion. Left upper quadrant pain along with fever, nausea, vomiting and anorexia may be present in combination [14]. Sarr and Zuidema suggested the triad of fever, left upper quadrant pain and a tender mass in patients with splenic abscess [15]. Pleural involvement along the diaphragm may lead to referred pain to the left shoulder with shortness of breath caused by sympathetic pleural effusion. Empyema may be associated once the effusion gets infected. In our case, the patient had empyema which was initially confirmed with aspiration followed by chest tube insertion. Pleural aspirate culture showed growth of *Streptococcus pyogenes* which itself is one of the rarest causes of empyema. The prevalence of Group A hemolytic Streptococcus (GAS)-associated pleural empyema is only 0.7% in patients with pleural empyema [16]. In the view of abscess in the lower pole of the spleen, we were not able to delineate if the patient had two different pathologies or the splenic entity caused the sympathetic empyema. Whatever the reason may be, the clinical improvement with the antibiotics that was initiated for the pleural culture organism *Streptococcus pyogenes,* improved the splenic entity and assumptions could be made that the two pathologies would be related to one another though the splenic aspirate culture was sterile.

Laboratory analysis usually show leukocytosis with left shift in splenic abscess. X-Ray Chest might show elevated left hemidiaphragm along with extra luminal air with features of pleural effusion which is evidenced as in our case. Ultrasonography (USG) abdomen might show enlarged spleen along with poorly defined hypoechoic or cystic lesion. CECT images demonstrate typical exhibition of a rim enhancement at the outside facing of the abscesses’ wall. The inside-facing portions of the wall usually show less-enhancing or non-enhancing components, that represent fibrous and proteinaceous material. The splenic abscess usually appears inhomogeneous with density values ranging from 20 to 40 HU. Gas formation within the abscess if encountered suspects the pyogenic lesion [17]. USG/CT guided aspiration confirms the diagnosis.

Treatment includes high dose broad spectrum antibiotics till the further diagnostic and therapeutic approaches are being considered. Percutaneous aspiration or drainage might be considered in patients with unilocular or discrete walled abscess with liquid component within [18]. This approach is carried out especially in critically ill patients or young immunocompetent patients as an attempt to preserve the spleen. A success rate of 50% has been mentioned in a series from Korea where the authors reviewed 18 patients with splenic abscess from November 1993 to December 2008 [19]. Open surgical drainage of abscess or splenectomy is mandated if the less invasive approaches like percutaneous aspiration or drainage with antibiotics fail. Furthermore, laparoscopic splenectomy has been a promising alternative to the open method, with faster recovery and short hospital stays [20].

## 4 Conclusion

Splenic abscess along with chest involvement should be considered in alcoholic patients who present with pain left hypochondrium with respiratory symptoms. Broad spectrum antibiotics along with percutaneous aspiration of splenic abscess if the lesion is unilocular along with tube thoracostomy is the treatment of choice in patients with pleural empyema complicated by splenic abscess.

## Declaration of Competing Interest

The authors report no declarations of interest.

## Funding

This case report did not receive any specific grant from funding agencies in the public, commercial, or not-for-profit sectors.

## Ethical approval

Ethical approval was not mandatory for publication of case reports as per the institutional policy.

## Consent

“Written informed consent was obtained from the patient for publication of this case report and accompanying images. A copy of the written consent is available for review by the Editor-in-Chief of this journal on request”.

## Author contribution

Design and Idea: Suman Baral, Raj Kumar Chhetri.

Drafting: Suman Baral.

Final Revision: Suman Baral, Raj Kumar Chhetri, Milan Gyawali, Neeraj Thapa.

## Registration of research studies

NA.

## Guarantor

Suman Baral.

## Provenance and peer review

Not commissioned, externally peer-reviewed.
